# Approaches to SARS-CoV-2 and other vaccinations in children with a history of multisystem inflammatory syndrome (MIS-C): An international survey

**DOI:** 10.3389/fped.2022.1030083

**Published:** 2022-11-09

**Authors:** Francesca Minoia, Federica Lucioni, Merav Heshin-Bekenstein, Sebastiaan Vastert, Christoph Kessel, Yosef Uziel, Lovro Lamot, Nicolino Ruperto, Marco Gattorno, Claudia Bracaglia, Natasa Toplak

**Affiliations:** ^1^ Pediatric Immuno-Rheumatology Unit, Fondazione IRCSS Ca’ Granda Ospedale Maggiore Policlinico, Milan, Italy; ^2^ Dana Dwek Children's Hospital, Tel Aviv Medical Center, Tel Aviv University, Tel Aviv, Israel; ^3^ Division of Pediatric Rheumatology and Immunology, Wilhelmina Children's Hospital, University Medical Center Utrecht, Utrecht, the Netherlands; ^4^ Department of Pediatric Rheumatology and Immunology, University Children's Hospital Muenster, Muenster, Germany; ^5^ Meir Medical Center, Sackler School of Medicine, Tel Aviv University, Kfar Saba, Israel; ^6^ University Hospital Center Zagreb, University of Zagreb School of Medicine, Zagreb, Croatia; ^7^ UOSID Centro Trial, IRCCS Istituto Giannina Gaslini, Genoa, Italy; ^8^ Center for Autoinflammatory Diseases and Immunodeficiencies, IRCCS Istituto Giannina Gaslini, Genoa, Italy; ^9^ Division of Rheumatology and Laboratory of Immuno Rheumatology, IRCCS Ospedale Pediatrico Bambino Gesù, Rome, Italy; ^10^ Department of Pediatric Allergology, Rheumatology and Clinical Immunology, University Children's Hospital, University Medical Centre, MF, UL, Ljubljana, Slovenia

**Keywords:** vaccination, multisystem inflammatory syndrome, pediatric inflammatory multisystem syndrome, MIS-C, SARS-CoV-2, COVID-19

## Abstract

**Background:**

Following the Coronavirus Disease-19 (COVID-19) pandemic outbreaks, the hyperinflammatory condition termed Multisystem Inflammatory Syndrome in Children (MIS-C) became a healthcare issue worldwide. Since December 2020 the mRNA vaccine against SARS-CoV-2 has become available with a good safety profile. However, evidence regarding safety and vaccination strategies in children with previous MIS-C is still lacking. The aim of our study was to investigate the current approach of international centers to anti-SARS-CoV-2 and other vaccinations in children with a history of MIS-C.

**Methods:**

Physicians who care for patients with MIS-C were invited to anonymously complete a 15-question, web-based survey. The survey was open from October 6 to December 31, 2021.

**Results:**

A total of 290 replies from 236 centers in 61 countries were collected. Most respondents (86%) were pediatric rheumatologists. The anti-SARS-CoV-2 vaccine was available in 85% of the countries. Sixty-seven centers (28%) in 22 countries already vaccinated MIS-C patients without adverse reactions in most cases (89%). Six reported complications: 2 not specified, 3 mild symptoms and 1 reported a MIS-C-like reaction. Most centers (84%) favored vaccinating MIS-C patients against SARS-CoV-2, after 3–6 months (40%), 6–12 months (52%) or >12 months (8%). The survey revealed broad heterogeneity of responses among healthcare providers within the same country and within the same center. The variable with the greatest impact on the decision not to vaccinate MIS-C patients was the current lack of evidence (51%), followed by patient/parent objection (40%). The most relevant parameters in the vaccination strategy were time from MIS-C episode (78%), immunosuppressive treatment (35%), SARS-CoV-2 serologic status (32%), and MIS-C features (31%). Almost all centers favored continuing regular vaccination with non-live (99%) and live (93%) vaccines; however, with high variability in suggested timelines.

**Conclusion:**

To date, the experience of the international pediatric rheumatology community in vaccinating MIS-C patients against SARS-CoV-2 is overall reassuring. However, lack of evidence causes broad heterogeneity in vaccination strategy worldwide.

## Introduction

Although SARS-CoV-2 infection in the pediatric population is usually asymptomatic or mildly symptomatic (1), some previously healthy children might develop a severe, hyperinflammatory condition, termed multisystem inflammatory syndrome in children (MIS-C) or pediatric inflammatory multisystem syndrome (PIMS), 2 to 6 weeks after SARS-CoV-2 infection (2). MIS-C is rare, affecting approximately one in 3,000–4,000 SARS-CoV-2 infections in unvaccinated children (3). Nonetheless, following the COVID-19 pandemic outbreaks, it rapidly became a healthcare issue worldwide, frequently requiring ICU admission and intensive treatments.

In December 2020, the US Food and Drug Administration and the European Medicines Agency approved the first mRNA anti-SARS-CoV2 (BNT162b2, Pfizer-BioNTech) vaccine for adults and adolescents from 16 years of age. In May 2021, this indication was extended to children from 12 years of age. Although the BNT162b2 vaccine has a very good safety profile in adults and children (4–6), data regarding safety and vaccination strategies in children with previous MIS-C are still limited (7, 8). Although rare, hyperinflammatory adverse events following vaccination with BNT162b2 have been reported, such as myocarditis (9–12) and MIS-C-like features (13–16), causing apprehension in vaccinating children with a history of MIS-C, given the hypothetical risk of recurrent hyperinflammation.

Against this background, as a joint effort of the Macrophage Activation Syndrome/systemic Juvenile Idiopathic Arthritis (MAS/sJIA) and the Vaccination Working Parties (WP) of the Pediatric Rheumatology European Society (PReS), we conducted a qualitative web-based international survey to investigate the approach to SARS-CoV-2 and other vaccinations among children with a history of MIS-C.

## Materials and methods

The core team of MAS/sJIA and Vaccination WP of PReS contributed to developing a 15-question web-based survey (Appendix). Most of the questions were multiple-choice answers with free text allowed for specific fields. Center, country, and specialty of participants were collected, together with an estimation of the number of MIS-C patients treated in each center. The experience of MIS-C patients who were re-infected by SARS-CoV-2 and subsequently presented a flare was also investigated. Physicians were asked about their current vaccination strategy for patients with previous MIS-C and to provide the most important variables affecting their decision-making process. Specifically, regarding anti-SARS-CoV-2 vaccination, participants were asked about the availability of the vaccine in their country, whether they have already vaccinated patients with a history of MIS-C, to provide an estimate of the number of patients vaccinated in each center and a description of adverse events. The survey was intentionally designed not to collect any identifiable clinical or demographic data from any specific patient and, due to that reason, ethical approval was not required, according to international and local regulations. Number of patients responses were evaluated as by center and checked to avoid duplicate patient entries. In case of disagreement on the number of patients reported by different physicians from the same center, the form with the later compilation date was recorded as the most up-to-date. Responses were collected voluntarily and anonymously. IP addresses were checked to guarantee unique participation.

The survey link was distributed through the main pediatric rheumatology international networks, including PReS, Emerging Rheumatologists and Researchers (EMERGE), Pediatric Rheumatology International Trial Organization (PRINTO) and the International Society of Systemic Auto-Inflammatory Diseases (ISSAID). The official involvement of the Childhood Arthritis and Rheumatology Research Alliance (CARRA) was not possible, due to the mandatory requirement of Institutional Review Board approval, which was unfeasible for time reasons. However, the link to the survey was forwarded also to North American physicians by personal email contacts and specialized email list-servers, thus North American centers which didn't need ethical approval according to local legislation, were involved in the project. Data were collected from October 6 to December 31, 2021.

Quantitative data are presented as median and interquartile range, categorical data are presented as absolute numbers and percentages.

## Results

**Figure 1:**
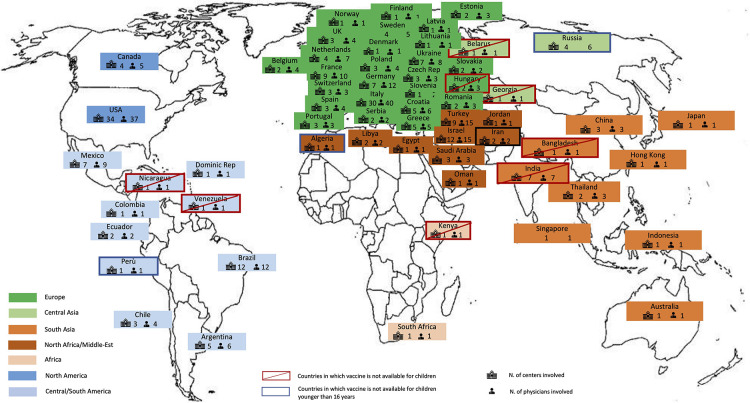
World map showing the 61 countries that participated to the survey, including the number of physicians, the number of centers involved, the countries in which the vaccination was not available for children or for individuals younger than 16 years.

Globally, 290 replies from 236 centers in 61 countries were collected (Figure 1). Most respondents (86.1%) were pediatric rheumatologists, while general pediatricians (5.2%), pediatric immunologists (3.1%), pediatricians from infectious disease departments (2.1%) and pediatric cardiologists (0.7%) also participated. The number of patients with MIS-C treated in each center varied greatly. Large and small centers participated in the survey: 24% treated less than 5 patients with MIS-C, 32% and 23% treated 5–20 and 20–50 patients, respectively, and 21% treated more than 50 patients.

At the time the survey was active, the anti-SARS-CoV-2 vaccine was available in 85% of the countries included. The vaccine was available for children 12 years of age and older in most of the countries, while in Algeria, Iran, Peru and Russia, approval was still limited to individuals from age 16 years only. In Bangladesh, Belarus, Egypt, Georgia, Hungary, India, Kenya, Nicaragua and Venezuela, the anti-SARS-CoV-2 vaccine was not available for children (Figure 1).

**Figure 2:**
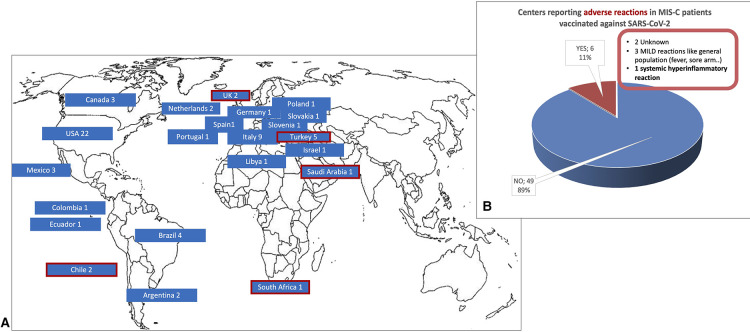
(**A**) The 67 centers from 22 countries that had already vaccinated patients with a history of MIS-C against SARS-CoV-2 and (**B**) reported adverse events*. In (**A**) the 5 countries in which 6 centers reported adverse events are circled in red. (*data available from 55 centers).

As shown in Figure 2, 67 centers (28%) in 22 countries (36%) already vaccinated patients with a history of MIS-C against SARS-CoV-2 at the time of the survey. Most vaccinated fewer than 5 patients (52%), 29% vaccinated 5 to 10 patients, and 20% reported to have already vaccinated more than 10 patients. Anti-SARS-CoV-2 vaccination was uneventful in most cases (89% of centers). Complications were reported by 6 centers: 3 reported mild symptoms common after vaccination in the general population (e.g., transient fever and sore arm), 2 did not specify the adverse events, and one reported a hyperinflammatory systemic reaction (Figure 2). Patients with a history of MIS-C who experienced SARS-CoV-2 re-infection were seen in 15% of centers and two reported a MIS-C flare.

Most physicians (84%) were in favor of vaccinating MIS-C patients against SARS-CoV-2. The 46 participants who disagreed did not have any specific background information, either geographically or in terms of subspecialty in common. Among them, only a quarter reported specific local recommendations against. Responses regarding the ideal time for vaccination varied greatly: 40% of participants suggested an interval of 3–6 months from the acute episode, 52% an interval of 6–12 months and 8% would have waited more than 12 months. Notably, in 50% of countries with more than one center involved in the survey, participants within the same country expressed opposing opinions regarding the intention to vaccinate MIS-C patients against SARS-CoV-2, and in 86% disagreed on the ideal vaccination time schedule. Furthermore, almost a quarter of physicians working in the same center expressed differing opinions on the indication to anti-SARS-CoV-2 vaccination.

**Figure 3:**
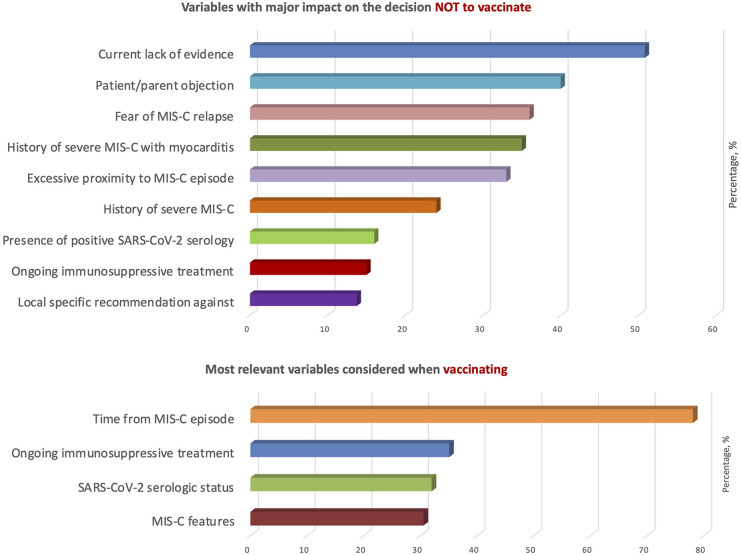
Variables with a major impact in the vaccination strategy against SARS-CoV-2 in patients with a history of MIS-C.

Variables with a major impact in the vaccination strategy against SARS-CoV-2 in patients with a history of MIS-C are highlighted in Figure 3. The variable with the greatest role in the decision not to vaccinate was the current lack of evidence (51%), followed by patient/parent objection, fear of MIS-C relapse and a history of severe MIS-C with myocarditis. The most relevant parameters considered in the vaccination strategy were time from MIS-C episode (78%), ongoing immunosuppressive treatment (35%), SARS-CoV-2 serologic status (32%) and MIS-C features (31%), especially severe cardiac involvement. Other relevant variables suggested by participants were the rate of SARS-CoV-2 infection in the region at the time of vaccination and previous treatment with high-dose intravenous immunoglobulins (IVIG).

Almost all physicians favored continuing the regular vaccination schedule, both with non-live (99%) and live (93%) vaccines. Regarding non-live vaccines, 43% of participants did not recommend a specific interval after the acute episode, while an interval of at least 3, 6 or 12 months was suggested by 33%, 17% and 6% of participants, respectively. Time schedules proposed for live vaccines were even more varied, given the potential impact of previous (IVIG) and ongoing (steroids and immunosuppressants) treatments: most participants suggested a delay of at least 3 (18%), 6 (24%) or 12 months (22%) after the acute episode, while almost a third did not specify a time recommendation.

## Discussion

International consensus on vaccination against SARS-CoV-2 in patients with a history of MIS-C is hampered by a lack of evidence both on safety and efficacy. At the time of the survey, data on tolerance of anti-SARS-CoV-2 vaccine in patients with a previous diagnosis of MIS-C were unavailable and uncertainty regarding the pathogenesis of MIS-C led to hesitancy in vaccinating. Indeed, 36 physicians from 21 countries reported local recommendations against anti-SARS-CoV-2 vaccination in patients with previous MIS-C at the time of our survey.

Recently, a multi-center cohort study of 63 children with a history of MIS-C and eligibility for vaccination described 15 patients (24%) who received anti-SARS-CoV-2 vaccination at a mean of 6.3 months from MIS-C episode without developing hyperinflammation, myocarditis, or re-occurrence of MIS-C (7). In an international survey involving 83 health care professionals (mostly general pediatricians), Hoste et al. reported 273 children with previous MIS-C (15.6% of eligible patients) who were vaccinated against SARS-CoV-2 worldwide. Although no individual data were collected in the context of that survey and most of the respondents did not provide specific data on adverse events, no MIS-C flare or any other inflammatory condition were observed (8). The survey data reported here covered 61 countries worldwide, involving 290 physicians (mostly pediatric rheumatologists) and highlighted the favorable opinion of the global pediatric rheumatologist community on vaccination against SARS-CoV-2 in children with previous MIS-C. The small percentage of centers (28%) that already experienced vaccination against SARS-CoV-2 in our study could be partially explained by the unavailability of the vaccine for children less than 12 years old at the time of the investigation. Similar to Hoste and colleagues, although no specific individual data were collected in our survey as well, all centers reported uneventful or mild and common adverse events (transient fever and sore arm) after SARS-CoV-2 vaccination, except for one that reported a MIS-C like flare. However, the lack of specific data regarding this single episode, such as interval from vaccination and epidemiological context, makes a clear cause-effect correlation impossible to define.

The risk of a reoccurrence of MIS-C after a second SARS-CoV-2 infection has not been established yet. Among the 26 centers (15%) that observed SARS-CoV-2 re-infection in children with a previous diagnosis of MIS-C, two reported a subsequent MIS-C flare. The role of vaccination in preventing MIS-C has been supported by several studies. BNT162b2 (Pfizer-BioNTech) vaccination was associated with a lower MIS-C incidence among adolescents in France (17). Vaccine effectiveness against MIS-C after the delta variant was estimated as 91% in children ages 12–18 years old in a US cohort (18) and 94% for children ages 5–17 years in a prospective nationwide study in Denmark (19). However, vaccine efficacy in preventing MIS-C related to new variants still has to be ascertained, as MIS-C in adolescents following exposure to the omicron variant was observed despite 3 doses of BNT162b2 vaccine (20).

The survey revealed wide heterogeneity of responses among participants within the same country and within the same center. In half of the countries with more than one center involved participants expressed opposing opinions regarding the intention to vaccinate MIS-C patients against SARS-CoV-2. Moreover, in more than 80% of cases, there was disagreement regarding the ideal vaccination time schedule, not only for SARS-CoV-2 but also for regular live vaccines. This result, together with the significant impact of the current lack of evidence in the decision-making process, highlights the urgent need for widely agreed recommendations, based on large, prospective international cohorts (21).

The current study should be interpreted in the light of some limitations. Due to its nature, in which no identifiable patient data were collected, the results cannot be used to estimate the incidence of adverse events or to define cause-effect correlations. Furthermore, results are limited to the experience of the participants and to the time the survey was conducted; moreover, a recall bias can't be completely excluded. Many other factors, such as the course of the pandemic, new variants of SARS-CoV-2, the vaccination rate among younger children and the development of new types of anti-SARS-CoV-2 vaccines could affect further vaccination strategies. In any case, the accumulation of real-world evidence will allow establishing the safety and efficacy of SARS-CoV-2 vaccination on MIS-C patients.

In conclusion, when vaccinating patients with a history of MIS-C against SARS-CoV-2, the experience reported by the international pediatric rheumatology community to date is overall reassuring. However, lack of evidence still prevents harmonization of the vaccination strategy, worldwide.

## Data Availability

The raw data supporting the conclusions of this article will be made available by the authors, without undue reservation.

## Appendix

**Current approach to COVID-19 and other vaccinations in previous MIS-C/PIMS patients: a MAS/sJIA and Vaccination WPs survey**

(1) Which is your country of practice? _________________
(2) Which is your center of practice? _________________
(3) Which is your field of expertise?
  □ General pediatrics
  □ Pediatric infectious disease
  □ Pediatric rheumatology
  □ Pediatric emergency care
  □ Pediatric cardiology
(4) How many patients with MIS-C/PIMS have been treated in your center?
  □ <5
  □ 5–20
  □ 20–50
  □ >50
(5) Is anti-SARS-CoV-2 vaccine available for children in your country?
  □ Yes, for children >16 years old
  □ Yes, for children >12 years old
  □ No
(6) Have you vaccinated any children with previous MIS-C/PIMS with anti-SARS-CoV-2 vaccine in your center?
  □ Yes
  □ No
(7) If you answered yes to Question 6, how many children with previous MIS-C/PIMS did you vaccinate with anti-SARS-CoV-2 vaccine in your center?
  □ <5
  □ 5–10
  □ >10
  □ Not applicable
(8) If you answered yes to Question 6, did you observe any side effects after anti-SARS-CoV-2 vaccine in patients with previous MIS-C/PIMS?
  □ Yes (specify): _________________
  □ No
  □ Not applicable
(9) How many of your patients with previous MIS-C/PIMS got COVID-19 infection again?
  □ None
  □ <2
  □ 2–5
  □ >5
(10) If you answered yes to Question 9, how many had a relapse of MISC/PIMS?
  □ None
  □ <2
  □ 2-5
  □ >5
  □ Not applicable
(11) If you vaccinated or aim to vaccinate with anti-SARS-CoV-2 vaccine patients with previous MIS-C/PIMS, what would, in your opinion, be a reasonable timing for the 1st booster vaccination?
  □ 3–6 months after MIS-C/PIMS
  □ 6-–12 months after MIS-C/PIMS
  □ Later than 12 months after MIS-C/PIMS
  □ I will not vaccinate patients with previous MISC/PIMS
(12) Which variables would impact your decision *not* to vaccinate with anti SARS-CoV-2 vaccine a patient with previous MIS-C/PIMS? (multiple choices possible)
  □ Current lack of evidence
  □ Fear of relapse
  □ Patient/parent decision
  □ Local specific recommendation against
  □ Ongoing immunosuppressive treatment
  □ Presence of positive SARS-CoV-2 serology
  □ History of severe MISC/PIMS
  □ History of severe MISC/PIMS with myocarditis
  □ Very recent MIS-C/PIMS episode
  □ Other: ___________________________
(13) Which variables would you consider most relevant in your decision to vaccinate with SARS-CoV-2 vaccine a patient with previous MIS-C/PIMS? (multiple choices possible)
  □ Time from MIS-C/PIMS episode
  □ SARS-CoV-2 serologic status
  □ Ongoing immunosuppressive treatment
  □ Severity of MIS-C/PIMS disease course
  □ Other
(14) Would you consider continuing regular vaccinations with non-live vaccines, according to the national vaccination program, in a child with previous MIS-C/PIMS?
  □ Yes
  □ Yes, but not before 3 months after MIS-C/PIMS
  □ Yes, but not before 6 months after MIS-C/PIMS
  □ Yes, but not before 12 months after MIS-C/PIMS
  □ No
(15) Would you consider continuing regular vaccinations with live attenuated vaccines, according to the national vaccination program, in a child after the MIS-C/PIMS?
  □ Yes
  □ Yes, but not before 3 months after MIS-C/PIMS
  □ Yes, but not before 6 months after MIS-C/PIMS
  □ Yes, but not before 12 months after MIS-C/PIMS
  □ No
